# A Discussion Following the Reading of a Paper upon "Syphlitic Affections of the Mouth"

**Published:** 1898-06

**Authors:** H. H. Burchard

**Affiliations:** Philadelphia Academy of Stomatology.


ARTICLE II.

### A DISCUSSION FOLLOWING THE READING OF A PAPER UPON "SYPHLITIC AFFECTIONS OF THE MOUTH."

DR. H. H. BURCHARD, OF PHILADELPHIA ACADEMY OF STOMATOLOGY.

Dr. E. C. Kirk: Mr. Chairman, I wish first to express my appreciation of the very clear presentation that Dr. Burchard has made of this very interesting subject. I wish especially to commend his drawings illustrating the several lesions he has described, and, further, incidentally to call attention to the very great desirability of having drawings of this character made by a dentist. I think they are the first drawings of this character, either anatomical or pathological, in which the human teeth have been represented as human teeth. They evidently bear the impress of a man who knows something of tooth-formation. They show that they were drawn by a dentist.

My first professional acquaintance with syphilis occurred, I think, in 1877, in listening to a lecture on this subject by the late Professor Agnew; and I was impressed at that time with the very great importance of the ability in the practitioner to recognize the characteristic features of syphilitic lesions, especially because of the necessity for protecting one's self and one's patients from the horrible results which might occur from any mistake in regard to diagnosis in this matter. Later on, when I took dental lectures, the subject was again impressed upon my mind, and I think that, as a graduate, I left college fully impressed with the great importance of recognizing these conditions. But as I went on in practice I found very few cases, and in looking back over perhaps twenty years of experience in contact with people of all sorts and conditions, the number of syphilitic lesions that I have observed in the mouth has been remarkably few. I made a sufficiently careful study of the subject to be able to recognize secondary, tertiary, and primary manifestations of this disease in the mouth; but as I say, the cases that have come to me have been extremely few in proportion to many other disorders—much less than I had anticipated from the instruction I received at college and the feeling that I had absorbed in regard to it.

I have seen a number of cases of secondary lesions in the mouth, and they have been sufficiently described by the essayist. I think we could all remember cases of this character. The same with the tertiary lesions.

It was my good fortune at one time—and only one time in my career—to meet with a primary syphilitic lesion in the mouth; the case was of a peculiar interest to me. It has never been reported, and I am glad of the opportunity to report it this evening. There was a necrotic patch or area just above the gingival margin of the upper left cuspid tooth, just as though a half-rounded chisel had cut out the gum tissue and left a base which was clay-colored, having the general characteristics of a mucous patch, but the edges were indurated. There was no pain to speak of, and no other lesion of the soft tissues of the mouth, but on examination I found a marked induration of some of the submaxillary and cervical lymphatics. The condition had existed for about ten days. Because of the peculiar appearance of the necrotic patch, because of the history of the case, because of its general clinical characteristics, and because of the induration of the lymphatic glands, I suspected that it might be a case of primary chancre. It was not complete; it was crecentic in character, not fully rounded, but looked as if infection had taken place at the gingival margin, and because of its location of circumference could not be completed. I dismissed the case for the time being, sent for the gentleman who referred the case to me, and asked him, if possible, to endeavor to ascertain the previous history. The girl told me that she had injured this gum-tissue with a tooth-brush, and this sore followed. I learned afterwards that while she was employed as a domestic in a boarding-house she had utilized the tooth-brush of one of the boarders to perform her dental toilet, and, as he was suffering from secondary syphilitic lesions of the mouth, the virus had been conveyed in that way. It is the only case of primary syphilis of the mouth I have ever seen, and was interesting because of its history and the method of transmission.

We are instructed (as we have been in the paper this evening) as to the especial care necessary to protect not only our patients, but ourselves, from syphilitic infection in the use of instruments. I heartily agree with all that the essayist has said as to the necessity for the sterilization of instruments and the maintenance of an aseptic condition of everything that goes into the mouth; and it seems hardly necessary to emphasize that, because it is presumable that the general principles of antiseptic surgery should apply to all dental operations, without regard to their specifically infectious character.

The question, however, of the contagiousness of syphilis is one that has interested me greatly. I have on two occasions, in operating for syphilitic patients where the lesions of the secondary stage existed, by accident subjected myself to the chances of infection, and have been caused a great deal of subsequent anxiety because of the possibility of personal infection. On one occasion I wounded my index finger with the plugger-point, and thought I was certainly infected with syphilis, but by careful sterilization of the wound, and by promotion of free bleeding at the time, no ill results followed.

On another occasion, in scaling the tooth of a patient whose mouth was affected with mucous patches, a particle of tartar sprang out of the mouth, passed beneath my glasses, and struck the sclerotic. I washed out the eye with warm water, and never had any difficulty. I am not immune from this disoder, but it shows that syphilis is not as contagious as it may have been considered; and, as a matter of fact, I think Dr. John Musser (of the University of Pennsylvania) within two or three years has written an essay on this particular point, showing that there is ground for belief that the type of syphilitic infection in later years has undergone considerable modification—that is to say, it is not as virulent as it was in its earlier history. How far that is true I do not know.

As to the question of tertiary lesions of syphilis from a practical point of view, a case was referred to me recently of tertiary syphilis, with perforation of the hard and soft palate, not involving the distal border of the velum, but simply a large oval area of the hard and soft palate. The case referred to me at the University, with the request that an obturator or an appliance be made to close this opening, in order that the patient might be able to masticate food and swallow better. I found that the edges of this opening were very much indurated, that the disease was still in an active state, that the imflammatory process was still active, that there was quite a considerable amount of bleeding from the margins of the opening, and from the fact that a flat obturator introduced under these circumstances would be in itself an irritant, I advised a postponement of mechanical treatment of the spot until the tissues could be brought to a more healthy condition. I felt sure of my ground at the time, but in thinking it over I have thought perhaps I might have been in error, and that the temporary closing of the orifice might have aided in the process of healing by relieving the tissues from irritation by food substances taken in the mouth. I would be very glad to have the judgment of my colleagues as to whether an obturator ought to have been inserted or postponed, as I advised, until the tissues were well.

Dr. M. H. Cryer: I would like to thank Dr. Burchard for his paper, and to ask him a question as to whether the teeth on the lower row in the drawing were not caused by salivation before the formation of enamel. In regard to Dr. Kirk's question, as to inserting an obturator before the orifice was healed, I should certainly not recommend it. No matter how carefully constructed, it would be an irritant. The exudations from this sore would be conveyed to other portions in contact with the mucous membrane. I should much prefer deferring the placing of the obturator until the tissues were in better condition.

Dr. H. B. Roberts: I would like to ask Dr. Burchard if it is possible for syphilis of the mouth to continue for periods running into years without destructive processes going on afterwards. I had as a patient a child that I believed to be syphilitic—born so; it is now nearly twelve years old, has never been able to talk intelligibly, it cannot walk without assistance, and its mouth seemed almost a mass of white mucous patches, located first at one place, then at another. The child was under my care for three or four years; it always came in with the same condition, and yet there was never any destruction of the tissues other than the mucous patches.

The only other case that I came across was that of tertiary syphilis with an ulcer half-way through the tongue. I had not suspected syphilis. I referred him to a physician, and he afterwards came back again with his tongue in a more healthy condition, following the use of iodides.

The President: We have with us this evening a gentleman from the medical profession in the person of Dr. Schamberg. I am sure the society will be glad to hear from him.

Dr. J. F. Schamberg: If I may be permitted to make a few remarks, I will preface them by thanking Dr. Burchard for his very admirable paper, and by stating that I would endorse *in toto* all the remarks he has made.

There can be no doubt whatsoever as to the importance of syphilis in relation to dental surgery. The disease is so wide-spread that many cases must of necessity come to the hands of the dentist, and particularly now, inasmuch as it is the custom of physicians who are treating cases of s philis to send them, immediately upon the recognition of the disease, to a dentist for the purpose of putting the mouth into proper shape.

We are aware of the fact that caries of the teeth and stumps in the mouth predispose to oral conditions in syphilis, to mucous patches, and other forms of syphiltic infection, and therefore dentists will be very apt to have cases of this nature come under their charge.

In the early stages of syphilis the disease is greatly contagious; and I may add here that it is not only contagious when there are certain lesions in the mouth, such as the initial lesions, chancre, and mucous patches, but it is contagious through the saliva when there are no oral lesions. I believe that the view of eminent syphilographers is that the virus of the secretions of the body in the second or constitutional stage of syphilis is infective, the tears, the saliva, very often the secretions of the stomach, and others. Therefore, even though there be no lesions whatsoever in the mouth, there is a possibility of infection, and the very practical point that this brings up is the thorough sterilization of all dental instruments after treatment not only of suspicious mouths, but of all mouths, because no one knows whether it is a syphilitic mouth he is treating or not. There are many cases of syphilis to-day going about without any cutaneous manifestations whatever, without any systemic or oral manifestations, and yet they are just as capable of transmitting this disease as if they were covered with these lesions.

Syphilis is a protean disease, manifesting itself in various ways, and Dr. Burchard has well illustrated the chief types of this disease, as well as its relation to the mouth.

I can state, in relation to Hutchinson's teeth, that Mr. Hutchinson says that the characteristic Hutchinsonian teeth are the upper central incisors. When other teeth are infected, they may be syphilitic teeth, but they are not pathognomonic; and the features are that crescentic excavation upon the free border of the tooth, the discoloration of the tooth, and, shortly, what he calls the "pegged-shape" tooth. The free border is of much less breadth than the gingival or alveolar border.

As regards the secondary patches of the mouth, they are very commonly confounded with lesions of this cavity. A great many skin affections, such as herpes and other disorders, always have concomitant lesions in the mouth, and herpes soon breaks down the covering, rupturing and exposing to view a small reddish abrasion, which may be redily mistaken for a mucous path. However, this matter of diagnosis is not of vital interest to the dentist, inasmuch as the question is, Shall he sterilize or exert any particular care or not?

In the tertiary lesions of syphilis of the mouth we have the gummata and what results therefrom—the deep ulcers; and it may be stated in general that the tertiary lesions of syphilis differ from the secondary in their deeper destruction; they destroy more and go more deeply, involving not only the submucous tissue, but also the bone and cartilage. In connection with these ulcers, one may lead to other ulcers in the mouth. There are epithelioma and tubercular ulcers, and I will say that it is extremely hard to differentiate them.

Syphilis is an extremely important disease, and I should like to correct any impression that may exist as to its infrequency. It is a very frequent disease, and it may be stated advisedly that it is just as frequent in high social stations as it is in low, not only abroad, but in this country, and almost every day dentists are bound to have patients come into their offices who have or have had syphilis.

An oral difference which may come under notice of the dentist is the peculiar whitish discoloration of the tongue or buccal mucous membrane; it is termed leucoplakia buccalis, or mucous plaques, and other varieties; some have been termed para-syphilitic. They are not due directly to syphilis, but to the ulterior influences of this disease. They are in many cases due to an irritation of the stomach, and this is perhaps the most frequent form. There is a form of syphilis which people have had in which a precedent syphilis has had some etiological influence.

I thank the society for the courtesy extended to me in permitting me to make these remarks.

Dr. J. H. Gaskill: I would like to ask Dr. Burchard a question. I had a case of syphilitic origin which involved the pharyngeal muscles, the soft and hard palate, the nasal bones, and a portion of the maxillary bones. The bones of the nose and a portion of the maxillary bone had to be removed, allowing the soft tissues of the nose to fall in, and making the face very flat. This allowed the end of the nose to point upward, elongating the nostrils and giving the patient so bad a deformity of the face that I have made him a false nose. I would like to ask whether there is a possibility of the irritation from the attachment of that nose inducing a trouble of another kind. To get a firm hold for that artificial substitute, I have made hard-rubber prolongations into the nostrils, and also passed a hook back of the lip; the upper part is held in place by spectacle frames.

Dr. H. H. Burchard: The answers to Dr. Kirk's and Dr. Gaskill's remarks both come under one head. The conditions they describe belong to the lesions of tertiary syphilis. Where these tertiary lesions of syphilis are present they are regarded as a local disease. They have this peculiarity, that they are locally infective, have a disposition to travel, and run a phagedenic course, and it is with extreme difficulty that they can be checked. So that in relation to Dr. Kirk's case, where the lesions are only partially healed, I deem it advisable to defer prosthesis until the healing is completed, after a course of iodides.

In Dr. Gaskill's Case, the degree of pressure permissible depends largely upon the thoroughness of the eradication of syphilis. There is always danger of precipitating degeneration in syphilitic tissues, so that I should say, Be guarded in the amount of pressure and its kind for a long period. Certainly, if the patient had any evidence of syphilis remaining, there would be danger of deep breaking down of the tissue, caused by this kind of irritation.

In regard to Dr. Cryer's question as to whether the Hutchinson teeth, or the other types of teeth shown here, could be caused by mercury or not, the best evidence in this matter is that of Hutchinson himself. It is a curious thing that, while during this time the upper central and all of the incisors are in process of formation, these two teeth, as a rule, should be the only ones affected. In mercurial cases all these teeth are affected; for example, when half the crowns of the upper central incisors are affected, about one-third the crowns of the lateral incisors and the tips of the cuspids are affected, and a large portion of the lower incisors. But in syphilis the pathognomonic teeth are the upper central incisors; the lower incisors may be perfectly normal. That is usually regarded as the point of differentiation between the two. In syphilitic children who have had a course of mercury the teeth often show evidence of faulty enamel formation; they have deep transverse grooves running across the crown, but these teeth show no evidence of gross malformation.

Dr. Roberts' question I would refer to Dr. Schamberg. In hereditary syphilis, as in this case, the lesions about the mouth would not have come under the head of mucous patches; these latter fungating ulcers, which disappear with the other manifestations of secondary syphilis. It has been maintained that syphilis is a self-limited disease in the sense of having a definite course. If no mercury were administered during the primary or secondary stage, and if no iodides were administered prior to or during the tertiary stage, then syphilis would run a distinctive course, the mucous patches belonging to the secondary constitutional stage. But as to the question why these ulcers in Dr. Roberts' case persisted, Dr. Schamberg will furnish him the answer.

Dr. J. F. Schamberg: I should state that at times, in syphilitic individuals, even the administration of the iodides of mercury would not be sufficient to dissipate any lesions of that disease, provided there was marked malnutrition: At times the so-called therapeutic test is not worked. And if these ulcers be syphilitic and do not heal, and the patient be under large doses of iodides and mercurials, then he may be classed as belonging to this group, in which the general condition of the patient was such as to fail to give the treatment a proper hold and fail to stimulate the regeneration of the tissues.—*International Dental Journal.*

